# A Comparison of Postoperative Outcomes in Immediate Versus Delayed Reconstruction After Mastectomy

**Published:** 2015-09-30

**Authors:** Paymon Sanati-Mehrizy, Benjamin B. Massenburg, John M. Rozehnal, Nachi Gupta, Jonatan Hernandez Rosa, Michael J. Ingargiola, Peter J. Taub

**Affiliations:** ^a^Icahn School of Medicine at Mount Sinai, New York, NY; ^b^Department of Emergency Medicine; ^c^Division of Plastic and Reconstructive Surgery, Department of Surgery, The Mount Sinai Hospital, Icahn School of Medicine at Mount Sinai, New York, NY

**Keywords:** breast reconstruction, immediate, delayed, flap, implant

## Abstract

**Objective:** The objective of this study was to evaluate the frequency of various postoperative complications in patients undergoing either immediate or delayed breast reconstruction after mastectomy for malignancy. **Methods:** The ACS-NSQIP 2005–2012 database was queried for patients who underwent mastectomy for the treatment of breast malignancy. These mastectomy cases were then stratified, generating “mastectomy alone” and “mastectomy with immediate reconstruction” cohorts. Database analysis also identified “delayed-reconstruction” oncologic patients. All patients undergoing reconstruction were then stratified into the tissue expander/implant or flap-based reconstruction group. The frequency of postoperative complications was assessed. A multiplicative risk model was used to calculate the probability of postoperative complications after undergoing a mastectomy alone, followed by reconstruction on a different date. These values were compared with the frequency of postoperative complications in the “mastectomy with immediate reconstruction” cohort, and 1-sample binomial tests were performed to determine statistical significance. **Results:** A total of 49,450 cases that underwent either mastectomy alone (*n* = 30,226), mastectomy with immediately tissue expander/implant reconstruction (*n* = 13,513), mastectomy with immediate flap reconstruction (*n* = 2854), delayed tissue expander/implant reconstruction (*n* = 2047), or delayed flap reconstruction (*n* = 810) were identified. When compared with a delayed reconstructive model, immediate reconstruction after mastectomy was associated with increased flap or tissue expander/implant failure. However, delayed reconstructive modalities were associated with increased postoperative medical and surgical complications. Finally, in flap-based reconstruction, the incidence of return to the operating room was higher in delayed reconstruction than in immediate reconstruction. **Conclusions:** Awareness of complications associated with each reconstructive modality will allow both surgeons and patients to effectively decide upon reconstructive options.

Breast reconstruction was the fifth most common reconstructive procedure performed by American plastic surgeons in 2014.^1^ When performed after mastectomy in the setting of breast cancer, breast reconstruction allows for improved psychiatric and aesthetic outcomes without effect on oncologic safety.^2^^-^7 However, the optimum timing for breast reconstruction remains to be clearly identified.^8^^-^13 Previous literature has indicated a possible association between increased postoperative complications with immediate breast reconstruction and isolated mastectomy.^9^^,^^14^ However, other studies have indicated similar postoperative outcomes without increased morbidity in immediate reconstruction.^15^ Furthermore, few studies compare immediate and delayed reconstructive modalities and thus effective selection of reconstructive timing is currently limited. The objective of this study was to evaluate the frequency of various postoperative complications as impacted by patient comorbidities in those undergoing either immediate or delayed breast reconstruction after mastectomy for malignancy.

## METHODS

The ACS-NSQIP 2005–2012 database was queried via *CPT* (*Current Procedural Terminology*) and *ICD-9* (*International Classification of Diseases, Ninth Revision*) codes for patients who underwent mastectomy for the treatment of breast malignancy. These mastectomy cases were then stratified on the basis of concomitant procedures to identify whether immediate reconstruction was performed, generating “mastectomy alone” and “mastectomy with immediate reconstruction” cohorts. The database was additionally queried for isolated reconstructive breast procedures, with *ICD-9* codes indicating a history of malignant breast neoplasm, identifying a cohort of “delayed-reconstruction” patients. All patients undergoing reconstruction were then stratified on the basis of reconstructive modality, including tissue expander/implant (TE/I) reconstruction and flap-based reconstruction. The frequency of postoperative complications, including return to the operating room (OR), wound complications (superficial surgical site infections, deep surgical site infections, organ-space site infections, and wound dehiscence), medical complications (pneumonia, postoperative reintubation, ventilator support for >48 hours, deep venous thrombosis, pulmonary embolism, renal insufficiency, progressive renal failure, urinary tract infection, stroke, coma, peripheral neurological deficiency, cardiac arrest, myocardial infarction, bleeding requiring transfusion, and sepsis/septic shock), and device or flap failure was assessed. A multiplicative risk model was used to calculate the probability of postoperative complications after undergoing a mastectomy alone, followed by reconstruction on a different date. These values were compared with the frequency of postoperative complications in the “mastectomy with immediate reconstruction” cohort, and 1-sample binomial tests were performed to determine statistical significance.

## RESULTS

A total of 49,450 cases that underwent either mastectomy alone (*n* = 30,226), mastectomy with immediately TE/I reconstruction (*n* = 13,513), mastectomy with immediate flap reconstruction (*n* = 2854), delayed TE/I reconstruction (*n* = 2047), or delayed flap reconstruction (*n* = 810) were identified.

### Patient demographics

Patient demographics were compared to ensure validity of performing a multiplicative risk model as a theoretic combined risk for delayed reconstruction. When compared with patients undergoing delayed TE placement, patients undergoing isolated mastectomies were older, had higher body mass indexes (BMIs), had higher ASA (American Society of Anesthesiologists) scores, and had an increased history of diabetes, hypertension, and dyspnea on exertion. Patients undergoing delayed TE placement were marginally older and had an increased history of hypertension with similar other medical risk factors when compared with those who had immediate TE placement (Table 1).

Similar comparisons were made for the flap-based reconstructive modality (Table 2). Patients undergoing isolated mastectomies were older, had higher ASA scores, and had an increased history of diabetes, hypertension, and dyspnea on exertion when compared with patients undergoing delayed flap reconstruction. Patients undergoing delayed flap reconstruction, when compared with patients undergoing mastectomy with immediate flap reconstruction, were marginally older with higher ASA scores.

### Postoperative complications in TE/I reconstruction

When compared with the calculated risk of delayed TE/I reconstruction, immediate reconstruction using TE/I was associated with decreased medical complications (*P* < .0001) and decreased surgical complications (*P* < .0001) (Table 3).

### Postoperative complications in flap reconstruction

The incidence of surgical complications was significantly decreased for the immediate reconstruction group when compared with the delayed flap reconstructive approach (*P* < .0001) (Table 4).

### Incidence of complications stratified by patient variables

The incidence of complications was also stratified on the basis of patient presurgical comorbidities to identify if certain patients were at a particularly increased risk with either reconstructive modality.

#### Medical complications

While the risk of medical complication was higher among all patients undergoing delayed rather than immediate TE reconstruction, this risk was particularly notable in patients with diabetes, those older than 65 years, smokers, those with higher ASA scores, and those with hypertension. The calculated risk of medical complications in flap-based reconstruction was also overall higher in the delayed reconstructive model, yet the extent of this effect was not statistically significant in any of the patient subsets analyzed.

#### Surgical complications

The risk for surgical complications was higher among both delayed TE and delayed flap-based breast reconstruction, when compared with their immediate reconstructive counterparts. The difference in TE-based reconstructions was more notable for patients with a history of smoking. The difference in flap-based reconstruction was more notable in patients with higher ASA scores, hypertension, and BMI of more than 35.

#### Flap failure

Delayed reconstruction appeared to have a protective effect for flap failure. Patients with diabetes and with a BMI of more than 35 had a more notable protective effect than patients who did not have these comorbidities. This may be due, in part, to the low number of flap failures reported in the NSQIP database over this time period. A similar protective effect was not observed in TE/I failure, but TE/I failure rates were much lower than flap failure rates.

#### Return to the OR

In flap-based reconstruction, the incidence of return to the OR was higher with delayed reconstruction than with immediate reconstruction. While the risk for return to the OR was increased with delayed TE reconstruction, this finding was not statistically significant.

## DISCUSSION

Medical and surgical complications were generally decreased with immediate reconstruction when compared with delayed reconstruction for both TE/I and flap reconstructions. In particular, a link was seen in patients older than 65 years, patients with a BMI of 35 or more, patients with a history of hypertension or diabetes, smokers, and patients with an ASA score of greater than 3 for TE/I reconstruction. Dyspnea on exertion did not have a statistically significant effect for TE/I reconstruction timing. In patients with these risk factors, the surgeon might consider immediate reconstruction to be particularly beneficial in reducing complications.

For flap reconstructions, patients with ASA score of greater than 3, a history of hypertension, or BMI of 35 or more tended to benefit more for reduction in surgical complications. Diabetes, age greater than 65 years, and a history of dyspnea on exertion did not have statistical significance for reduced surgical complications. For patients undergoing flap reconstruction with the aforementioned risk factors, the surgeon might be more inclined to do an immediate reconstruction rather than delayed reconstruction to prevent surgical complications. These data do, however, suggest that delayed flap reconstruction was associated with fewer incidents of flap failure, more notably in smokers and patients with higher ASA scores. This may be due, in part, to the low number of flap failures reported in the NSQIP database; however, the surgeon should take this into account in those respective patient populations.

While delayed flap reconstruction was associated with a protective effect for flap failure, a similar protective effect was not noted in TE/I reconstructions. It should be noted, however, that TE/I failure rates overall were lower than flap failure rates.

Flap reconstructions were also generally associated with more medical and surgical complications than with TE/I reconstructions, which the surgeon should take into account when determining whether TE/I or flap reconstruction is more appropriate. Better postoperative care for flap reconstructions may also be beneficial.

Several limitations apply to this study, largely due to its design and data source. As a retrospective analysis, a certain level of selection bias certainly exists and control over patient variables is limited. However, the NSQIP database has been continually validated since its creation and the program has a number of auditing procedures to limit its selection bias. Furthermore, our analysis for postoperative complications is limited to a 30-day postoperative course. A number of complications, including TE/I failure, is often seen outside this time period. Considering these limitations, the large sample size afforded by this national database allows for analysis that often cannot be performed with smaller sample sizes, serving as a method for preliminary analysis to stimulate future prospective studies.

## CONCLUSIONS

There is little consensus on how soon after mastectomy reconstruction should take place. The NSQIP database queried from 2005–2012 suggests that the incidence of postoperative medical and surgical complications in TE/I and flap reconstructions was decreased in cases with immediate reconstruction when compared with delayed reconstruction. Furthermore, immediate reconstruction was associated with decreased rates of return to the OR. Patients with preoperative medical risk factors, such as increased age, higher ASA scores, hypertension, diabetes, smoking, and higher BMI tended to benefit more from immediate reconstructions. For flap failure, however, delayed reconstruction was associated with a protective effect, which may be due, in part, to the low number of reported flap failures. TE/I reconstructions overall had fewer medical or surgical complications than with flap reconstructions. The surgeon should take the patient's preoperative medical risk profile into account when determining whether immediate or delayed reconstruction would be more beneficial for the patient.

## Figures and Tables

**Table 1:** Patient demographics of TE/I reconstruction cohort*

Variables	Isolated mastectomy (*n* = 30,226)	Delayed TE (*n* = 2047)	*P* (isolated vs delayed)	Mastectomy + immediate TE (*n* = 13,513)	*P* (delayed vs immediate TE/I)
Age, mean ± SD, y	61.97 ± 13.49	52.48 ± 10.59	<.0001	51.4 ± 10.64	<.0001
BMI, mean ± SD	29.01 ± 7.33	26.73 ± 6.3	<.0001	27.01 ± 6.4	.065
ASA score, mean ± SD	2.37 ± 0.6	2.16 ± 0.51	<.0001	2.12 ± 0.53	.157
Smoking	14.17%	13.29%	.2769	13.71%	.632
Diabetes	14.25%	5.52%	<.0001	4.69%	.113
Hypertension	48.81%	27.70%	<.0001	24.10%	.0005
Dyspnea on exertion	8.93%	3.57%	<.0001	3.10%	.291
Race					
White	70.36%	82.36%	.0001	81.58%	.408
Black	11.75%	7.52%	.0001	6.78%	.233
Hispanic	1.03%	0.88%	.5663	0.61%	.203
Asian	4.89%	2.10%	.0001	3.02%	.026
Pacific Islander	1.47%	0.24%	.0001	0.41%	.348

*TE/I indicates tissue expander/implant; TE, tissue expander; BMI, body mass index; and ASA, American Society of Anesthesiologists.

**Table 2:** Patient demographics of flap-based reconstruction cohort*

Variables	Isolated mastectomy (*n* = 30,226)	Delayed flap (*n* = 810)	*P* (isolated vs delayed)	Mastectomy + immediate flap (*n* = 2854)	*P* (delayed vs immediate flap)
Age, mean ± SD, y	61.97 ± 13.49	53.61 ± 10.65	.0001	52.00 ± 9.62	<.0001
BMI, mean ± SD	29.01 ± 7.33	29.30 ± 7.19	.265	28.63 ± 6.46	.011
ASA score, mean ± SD	2.37 ± 0.6	2.29 ± 0.52	.000	2.15 ± 0.54	<.0001
Smoking	14.17%	10.70%	.007	10.70%	.982
Diabetes	14.25%	8.40%	<.0001	5.05%	.0005
Hypertension	48.81%	29.50%	<.0001	27.00%	.166
Dyspnea on exertion	8.93%	4.57%	<.0001	3.61%	.255
Race					
White	70.36%	76.23%	.000	73.37%	.111
Black	11.75%	13.18%	.233	11.46%	.202
Hispanic	1.03%	0.37%	.095	0.81%	.284
Asian	4.89%	2.09%	.000	3.26%	.11
Pacific Islander	1.47%	0.25%	.006	0.28%	.87

*BMI indicates body mass index; and ASA, American Society of Anesthesiologists.

**Table 3:** Incidence of complications after immediate and delayed TE/I breast reconstruction*

	Medical complications				Surgical complications			
	Immediate reconstruction	Delayed reconstruction	RR (CI) of delayed reconstruction	*P*	Immediate Reconstruction	Delayed Reconstruction	RR (CI) of delayed reconstruction	*P*
Overall	1.66%	3.06%	1.84 (1.4–2.43)	<.0001	4.09%	6.58%	1.61 (1.34–1.93)	<.0001
Risk stratified								
DM	1.10%	4.75%	4.3 (1.42–13)	.0292	8.36%	9.42%	1.13 (0.61–2.09)	.5875
Age ≥65 y	1.61%	4.19%	2.61 (1.33–5.12)	.0072	5.57%	6.26%	1.13 (0.69–1.84)	.5784
Smoking	1.51%	3.32%	2.2 (1.05–4.6)	.0002	5.78%	9.74%	1.69 (1.13–2.52)	.0126
ASA score ≥3	2.45%	4.78%	1.95 (1.21–3.16)	.008	7.27%	8.33%	1.15 (0.82–1.61)	.491
HTN	2.03%	3.52%	1.73 (1.06–2.84)	.038	5.99%	7.59%	1.27 (0.92–1.74)	.1727
BMI ≥35	2.31%	3.40%	1.49 (0.71–3.11)	.4778	10.69%	13.04%	1.22 (0.84–1.78)	.3521
DoE	2.39%	6.78%	2.84 (1–8.1)	.0564	6.92%	13.16%	1.9 (0.97–3.73)	.0593
	**Graft failure**				**Return to OR**			
	**Immediate reconstruction**	**Delayed reconstruction**	**RR (CI) of delayed reconstruction**	***P***	**Immediate reconstruction**	**Delayed reconstruction**	**RR (CI) of delayed reconstruction**	***P***
Overall	0.74%	0.49%	0.66 (0.34–1.26)	.261	7.43%	8.04%	1.08 (0.92–1.27)	.335
Risk stratified								
DM	1.42%	1.08%	0.76 (0.12–4.99)	.991	9.46%	7.29%	0.77 (0.38–1.57)	.562
Age ≥65 y	0.68%	1.16%	1.71 (0.5–5.82)	.754	7.67%	6.85%	0.89 (0.56–1.42)	.66
Smoking	1.51%	1.09%	0.72 (0.22–2.37)	.7992	11.02%	11.53%	1.05 (0.73–1.49)	.933
ASA score ≥3	1.03%	0.39%	0.38 (0.08–1.78)	.3816	9.33%	7.92%	0.85 (0.61–1.19)	.4292
HTN	1.20%	0.33%	0.27 (0.06–1.19)	.1141	9.13%	8.25%	0.9 (0.67–1.21)	.581
BMI ≥35	1.94%	0.68%	0.35 (0.07–1.82)	.2	12.01%	10.15%	0.85 (0.56–1.29)	.481
DoE	0.48%	0.22%	0.46 (0–73.38)	.554	6.92%	13.16%	1.9 (0.97–3.73)	.82

*TE/I indicates tissue expander/implant; RR, relative risk; CI, confidence interval; DM, diabetes mellitus; ASA, American Society of Anesthesiologists; HTN, hypertension; BMI, body mass index; DoE, dyspnea on exertion; and OR, operating room.

**Table 4:** Incidence of complications after immediate and delayed flap-based breast reconstruction*

	Medical complications				Surgical complications			
	Immediate reconstruction	Delayed reconstruction	RR (CI) of delayed reconstruction	*P*	Immediate reconstruction	Delayed reconstruction	RR (CI) of delayed reconstruction	*P*
Overall	8.76%	9.24%	1.05 (0.82–1.35)	.618	5.99%	10.09%	1.68 (1.31–2.17)	<.0001
Risk stratified								
DM	11.11%	17.09%	1.54 (0.77–3.07)	.274	13.19%	16.17%	1.23 (0.62–2.43)	.7111
Age ≥65	8.90%	9.92%	1.11 (0.58–2.15)	.9865	6.05%	7.76%	1.28 (0.6–2.72)	.623
Smoking	8.88%	9.48%	1.07 (0.5–2.26)	.928	10.53%	11.81%	1.12 (0.57–2.19)	.952
ASA score ≥3	13.07%	14.96%	1.14 (0.8–1.63)	.555	8.35%	12.70%	1.52 (1.01–2.29)	.0446
HTN	9.97%	13.45%	1.35 (0.92–1.98)	.1713	7.51%	13.20%	1.76 (1.17–2.64)	.0079
BMI ≥35	12.16%	11.84%	0.98 (0.59–1.62)	.989	11.56%	19.22%	1.66 (1.07–2.58)	.0337
DoE	6.80%	9.45%	1.39 (0.38–5.1)	.79	9.71%	14.42%	1.49 (0.54–4.06)	.7399
	**Flap failure**				**Return to OR**			
	**Immediate reconstruction**	**Delayed reconstruction**	**RR (CI) of delayed reconstruction**	***P***	**Immediate reconstruction**	**Delayed reconstruction**	**RR (CI) of delayed reconstruction**	***P***
Overall	2.87%	1.38%	0.48 (0.26–0.89)	.0218	10.44%	13.14%	1.26 (1.02–1.55)	.0322
Risk stratified								
DM	2.78%	0.20%	0.07 (0.01–0.63)	.919	12.50%	17.86%	1.43 (0.73–2.8)	.428
Age ≥65 y	2.85%	0.90%	0.32 (0.04–2.23)	.3483	9.96%	10.81%	1.08 (0.59–1.99)	.861
Smoking	3.29%	2.66%	0.81 (0.2–3.31)	.4947	13.16%	13.69%	1.04 (0.57–1.9)	.878
ASA score ≥3	3.31%	0.55%	0.17 (0.03–0.91)	.0214	13.86%	12.85%	0.93 (0.64–1.35)	.7845
HTN	4.02%	1.41%	0.35 (0.11–1.07)	.0624	14.12%	13.99%	0.99 (0.69–1.42)	.988
BMI ≥35	5.38%	0.22%	0.04 (0–1.41)	.0127	16.40%	18.67%	1.14 (0.75–1.73)	.6515
DoE	1.94%	0.22%	0.11 (0–122.47)	.784	12.62%	15.45%	1.22 (0.5–2.99)	.789

*RR indicates relative risk; CI, confidence interval; DM, diabetes mellitus; ASA, American Society of Anesthesiologists; HTN, hypertension; BMI, body mass index; and DoE, dyspnea on exertion; and OR, operating room.
